# Annotations

**Published:** 1910-12-31

**Authors:** 


### Annotations.

### The Houndsditch Murders.

Into the pros and cons of such vexed questions as the arming of the police force with firearms and the similar problems which the recent murder of three policemen by alien criminals has provoked, it is not necessary to enter in the pages of a professional journal. To the medical mind the ethnological and psychological aspects of the tragedy appeal as well as the heroic, and the desperate, characters of the constables and their assailants respectively. It is a most remarkable thing that a crime such as this, so foreign to the nature of the British burglar, should yet be so consonant with that of the alien immigrant from Poland, Italy, and other continental countries. Our own criminal classes contain men of scant scruple and degraded vices, to say nothing of a certain kind of courage. Yet it never occurs to them to proceed to such extremes of violence as these Houndsditch scoundrels adopted. In saying this it is perhaps wise to remember the story of Bill Sikes' crimes, which may possibly have been suggested to Dickens by some equal brutalities a couple of generations ago. But at least nowadays the metropolitan police force is rarely, if ever, shot down in this way by home-grown desperadoes. Those who are concerned for the future of the race and would close our doors to the riff-raff, morally and physically, of the Continental guttersnipes who pour in upon us, ascribe part of the evil to the relaxation by Lord Gladstone of the Aliens Act, which has converted that safeguarding measure practically into a dead letter. Whether this be so or not, it does seem as if the introduction of elements so absolutely lawless, so bestial, and so dangerous as these alien degenerates is a serious menace to that sane and practical genius which has always hitherto distinguished the British race, high and low, and has been one of the principal sources of the strength and solidarity of the British Empire.

### Public Health and Local Administration.

A number of matters of medical interest are dealt with in the thirty-ninth annual report of the Local Government Board, which has just been issued. Many of these matters demand a fuller and closer scrutiny than can be given them on this page, and we must therefore postpone their consideration to a later date. The number of cases of fever and diphtheria treated by the Metropolitan Asylums Board or receivable into their hospitals in 1909 was 25,003 and of small-pox twenty-one. The length of residence of patients who completed their recovery or died at the managers' town hospitals was, on an average, as follows: Scarlet fever, 61.7 days; diphtheria, 53.9 days; diphtheria (bacteriological), 31.7 days; enteric fever, 60.9 days; miscellaneous (mistaken diagnosis), 24.6 days. The maximum number of patients in these hospitals on any one day was 3,920, and the minimum 3,126. The case mortality from scarlet fever was 2.3 per cent., from diphtheria 9.4 per cent., and from enteric fever 11.9 per cent. A table is also given showing the aggregate number of notifications of each disease, the deaths, and the percentage of mortality to number of cases notified in London and 257 towns and in fifty-five port sanitary districts. The annual reports of medical officers of health continue to show improvement in the administration of the law relating to milk-supply, and in the condition of premises on which milk is handled. Over 150 local authorities have now made arrangements for the veterinary inspection of milch cows in their districts. In the majority of cases in which infectious disease was attributed to milk supply, the farmer or dairyman in question ceased voluntarily to supply milk when requested by the medical officer of health to do so. For this reason local authorities have in practice only had occasion to make a formal order prohibiting the supply of milk in a small number of cases. The number of persons on whom successful primary vaccinations were performed by the public vaccinators during the year ended September 29, 1909, was 432,829, of whom over four hundred thousand were infants under one year of age; in fact, nearly 50 per cent. of the children born in the year were vaccinated at the public expense. The number of applications for vaccine lymph received from public vaccinators and complied with during 1909 amounted to 66,775. The total number of samples of foods and drugs analysed during the year was 98,544, and the proportion of samples reported against was 7.5 per cent., which is a lower percentage than was recorded in any previous year. Legal proceedings were instituted against the vendors in 3,235 cases, penalties being imposed in 2,408 cases.

### The late Professor Huchard.

Professor Henri Huchard, member of the Paris Academy of Medicine, honorary physician to the Necker Hospital and one of the best-known specialists for diseases of the heart in France, died on December 11, at the age of sixty-six. Born at Auxon (Aube) in 1844, he became interne to the Paris hospitals in 1867, contracting while on duty in the small-pox wards a severe attack of the disease which nearly cost him his life. His devotion was recognised by his being made a member of the Legion of Honour. In 1875 he received his doctor's degree, and three years later was appointed physician to the Paris Hospitals. He quickly specialised in diseases of the heart and blood-vessels, and in 1884 won the Godard prize, and in 1893 the Montyon and Chateauvillard prizes, for work connected with his speciality. Although a professor without an official chair, his lectures were always well attended, and it is a matter for regret that the antiquated conditions upon which professorships are conferred should have deprived the French schools of his services. As a medical journalist he was well known, especially in connection with his plans for the reform of medical education. Alike friends and enemies—for a man of his sincerity and plain speaking was bound to have some of the latter—deplore his loss, the loss of one who by his own unaided genius was at once a great physician and a great teacher.

